# Nursing care for women with gynecologic cancer receiving radiotherapy: current updates

**DOI:** 10.4069/kjwhn.2023.12.11

**Published:** 2023-12-28

**Authors:** Hyesung Moon

**Affiliations:** Department of Nursing, Ewha Womans University Mokdong Hospital, Seoul, Korea

## Background

Approximately 36% of all cancer patients in Korea receive radiotherapy, a key modality of cancer treatment alongside surgery and chemotherapy. Diverse therapeutic techniques have resulted from advances in radiological technology and treatment devices, contributing to the improvement of the quality of treatment and patients’ quality of life.

According to 2022 data from the Korea Institute of Radiological Medical Sciences, 7.8% of female patients who underwent radiotherapy in 2019 received treatment for gynecologic cancer. Notably, approximately 98% of brachytherapy procedures were conducted to treat gynecologic cancer [1]. Radiotherapy for gynecologic cancer, which can involve both teletherapy and brachytherapy, plays a pivotal role in improving treatment outcomes because it targets not only the early stages of the cancer but also advanced lesions for radical, adjuvant, and palliative purposes. This article explores recent developments in radiotherapy, with a particular focus on radiotherapy for gynecologic cancer, and discusses acute and chronic adverse events that may occur during treatment, as well as interventions.

## Latest trends in radiotherapy

**Figure 1.**
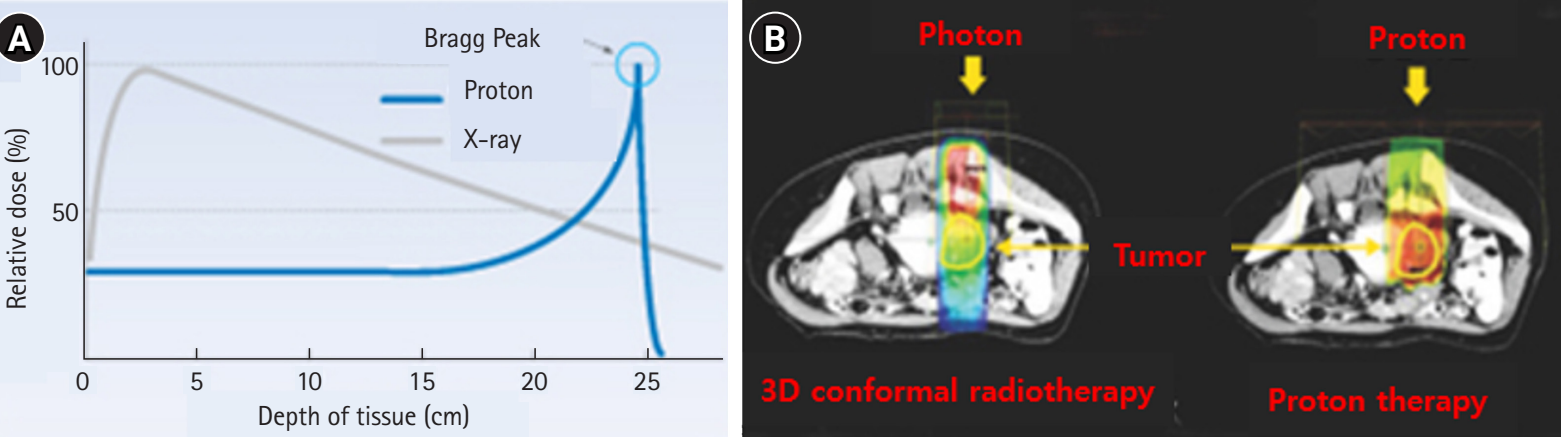
(A) Particle vs. photon beam dose penetration. (B) Particle vs. photon beam dose distribution. Source: Ministry of Health and Welfare (https://www.mohw.go.kr/synap/doc.html?fn=20070410094312467018_1.hwp&rs=/upload/result/202312/).

Radiotherapy aims to maximize treatment effectiveness and minimize side effects by primarily irradiating tumor tissues and limiting radiation exposure to the surrounding normal tissues. Radiotherapy technology has advanced dramatically over the last 20 years, and the effectiveness of concomitant chemoradiotherapy treatment has been proven in a substantial number of studies as presented in a recent systematic review [2]. Radiotherapy initially involved treatment in a flat, two-dimensional (2D) plane based on 2D imaging centered around tumors. Subsequently, three-dimensional (3D) conformal radiotherapy became available; this method models tumors and the surrounding tissues in three dimensions using computed tomography and magnetic resonance imaging, enabling more precisely targeted treatment. High-precision radiotherapy procedures, such as intensity-modulated radiotherapy (IMRT), respiratory-gated radiotherapy, image-guided radiotherapy, and stereotactic radiotherapy, emerged in the 2000s. Active research is currently underway in the field of concomitant chemoradiotherapy, which combines radiotherapy with cancer immunotherapy [3]. Furthermore, particle therapies using protons and heavy ion particles, which are recognized for their higher treatment effectiveness and fewer side effects compared to traditional high-energy X-ray therapies listed above, have gained attention in recent years [4,5] (Figure 1). Both therapies utilize subatomic particles, and their utility is based on the ‘Bragg peak,’ a phenomenon where particles penetrate normal tissues in the human body and emit radiation energy only at specific depths where tumor tissues are found [6] (Figure 1).

However, cost-effectiveness should be carefully considered when choosing a treatment modality, since radiotherapy using X-rays has favorable treatment outcomes for gynecologic cancer, while particle therapy is currently offered only at three medical institutions in Korea and heavy ion particle therapy is not covered by insurance.

**Figure 2.**
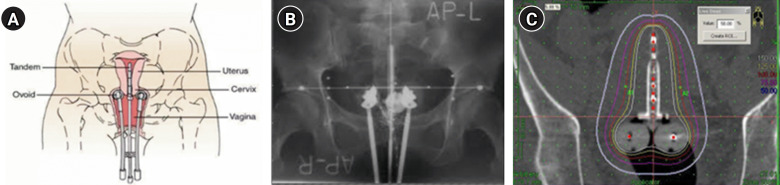
(A) Diagram showing the positions of the tandem and ovoid in the uterus and vagina from the front. Source: About Cancer (https://www.aboutcancer.com/intracavitary_radiation_treatments.htm). (B) Anterior-posterior treatment planning X-ray of the tandem and ovoid applicator. (C) Typical anterior-posterior isodose distributions of a high-dose-rate tandem and ovoid applicator.

Gynecologic cancer is unlike other malignancies in that it often requires brachytherapy, which involves intravaginal insertion of devices, and the placement of isotopes close to the lesion for treatment, in combination with teletherapy. The advantages of brachytherapy include the delivery of a high dose of radiation due to the proximity of the equipment to the treatment area and the minimization of effects on normal tissues in the bladder and rectum [7,8] (Figure 2). Data reported in 2021, however, showed that the availability of brachytherapy decreased from 84% in 2005 to 78% in 2013 in Korea and the number of medical institutions that offer brachytherapy also declined from 65% in 2006 to 36.8% in 2014 [9]. Since it is impossible to maintain facilities for brachytherapy available due to low medical reimbursements and challenges in equipment management, patients are often referred to other institutions.

## Patient care by radiotherapy for gynecologic cancer

Unlike chemotherapy, most symptoms related to radiotherapy occur locally in the treatment area and are affected by the method, area, dose, and duration of treatment, as well as the patient’s general condition. In general, radiotherapy for gynecologic cancer involves irradiating the pelvis, and the treatment area can be expanded to the upper abdomen if the paraaortic lymph nodes are included in the treatment. Gastrointestinal symptoms, micturition, and genital disorders can occur due to the treatment. While most symptoms improve substantially within 6 months after the end of treatment, some patients suffer prolonged discomfort due to the persistence of these symptoms [10].

## Gastrointestinal complications

Gastrointestinal symptoms caused by radiotherapy for gynecologic cancer include diarrhea, nausea, vomiting, tenesmus, and rectal bleeding. Approximately 30% of patients who undergo pelvic radiotherapy experience acute enteritis accompanied by diarrhea. In 10% of these patients, the symptom persists even after 5 years after treatment completion [10]. A study that compared existing 3D conformal radiation therapy (CRT) and IMRT reported that grade 3 or more severe diarrhea [11] occurred significantly more frequently in the 3D CRT group than in the IMRT group (30.6% vs. 5.6%) [12]. Therefore, IMRT has become the more widely used procedure. Common approaches to acute enteritis include fiber products, antidiarrheal agents, and the supply of fluids and electrolytes through intravenous hydration. For malabsorption due to chronic enteritis, the use of vitamin B12 and cholestyramine for bile salt malabsorption can be actively considered [13]. Anti-inflammatory agents, intestinal protectants, intestinal antimotility agents, and probiotics are generally used for acute radiation proctitis [14], and if rectal bleeding persists, endoscopic treatment, such as a sucralfate enema or argon-plasma coagulation, may be required [15,16].

## Genitourinary complications

The bladder and ureter are inevitably exposed to radiation due to their close proximity to organs affected by gynecologic cancer. In the GOG-99 study, low-grade genitourinary toxicity was reported in approximately 43% of patients following radiotherapy after endometrial cancer surgery [10]. Symptoms include frequent urination, dysuria, and rarely, hematuria. When these symptoms occur, urinalysis and culture tests are conducted to determine whether the patient has an infection, and medication is then prescribed. If infection is ruled out, ibuprofen and phenazopyridine may be helpful for frequency, and anticholinergics can be helpful for urgency [10]. If oral medications are not effective, the cystoscopic injection of botulinum toxin A can be attempted [17]. For hemorrhagic cystitis, which can occur chronically, laser fulguration of ectatic vessels, intravesical alum or formalin, or hyperbaric oxygen may be considered [18]. Urethral strictures, which may occur in less than 5% of patients, can be addressed by endoscopic dilation or stent placement. While vesicovaginal fistulas are typically managed through simple fulguration and catheter drainage, they sometimes require open surgical repair [19].

## Sexual dysfunction

**Figure 3.**
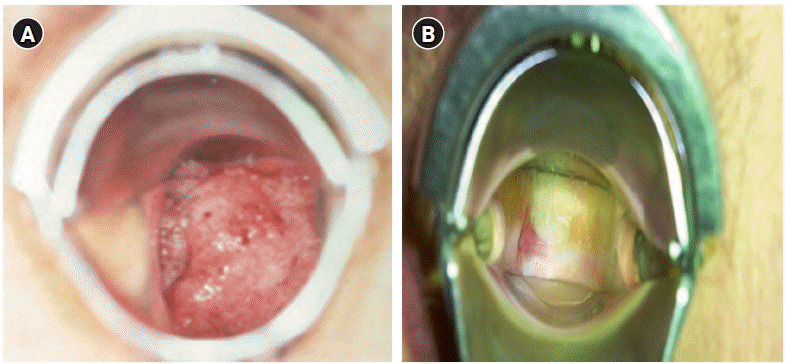
Comparison of the cervix before and after radiotherapy. (A) Cervical cancer IIb, pre-radiotherapy. (B) Five months after radiotherapy (external beam radiotherapy [50.4 Gy]+high-dose-rate brachytherapy [24 Gy]).

The most frequent complications experienced by gynecologic cancer patients who have undergone pelvic radiotherapy are vaginal stenosis and diminished ovarian function in premenopausal women. These complications can lead to challenges in sexual relationships due to dyspareunia and reduced vaginal discharge, resulting in negative effects on women’s quality of life. The incidence of vaginal strictures due to radiotherapy varies from 1.2% to 88%, depending on the patient’s personal characteristics, treatment method, and dose. Vaginal strictures occur in 50% or more of patients within 3 years after the completion of treatment [20] (Figure 3).

**Figure 4.**
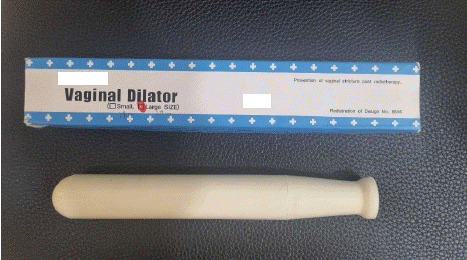
Vaginal dilator.

In general, patients undergoing pelvic radiotherapy often experience menopause within 6 months after completing treatment. To alleviate the symptoms of menopause, oral progesterone and/or estrogen with a serotonin-specific reuptake inhibitor can be administered. Vaginal dilators are commonly employed as a treatment for vaginal strictures. Patients are advised to use these dilators for a duration of 10 to 15 minutes, 2 to 3 times per week, for a period of 3 to 12 months posttreatment (Figure 4).

In contrast to the past, there is now a rising demand to address sexual issues alongside physical symptoms, with a growing interest in maintaining functionality. Therefore, several models are being proposed to aid in sexual assessment and interventions [21] (Table 1.).

## Hematologic toxicity

High-dose radiotherapy causes chronic myelosuppression and damage to the bone marrow microenvironment, potentially affecting the effectiveness of chemotherapy. A study reported that acute grade 3 (Common Toxicity Criteria version 2.0, National Cancer Institute) or more severe leukopenia occurred in 81% of patients undergoing cisplatin-based pelvic chemoradiotherapy, which included expanded coverage of the para-aortic lymph nodes or common iliac lymph nodes [22]. Hence, it is necessary to monitor the neutrophil, platelet count, and hemoglobin levels through weekly blood tests. Active interventions should also be implemented, including assessing the risk of infection, based on the severity of the condition.

## Dermatologic toxicity

Most skin reactions to gynecologic cancer radiotherapy are grades 1 to 2, but moderate or more severe skin reactions (grading criteria for radiodermatitis by the Radiation Therapy Oncology Group) are observed in up to 95% of patients with vulvar cancer [23]. Mild erythema may occur in the vulva, perineum, and inguinal and gluteal folds about 2 to 3 weeks after pelvic teletherapy, and this can be alleviated by using a topical moisturizer. Vulvar cancer patients often have white plaques covering their skin due to the overgrowth of *Candida*. Wearing loose-fitting, cotton clothing, avoiding heat, and using 1% hydrocortisone cream if pruritis is present may help alleviate this symptom. Late dermatologic effects include hyperpigmentation or hypopigmentation, as well as telangiectasis and textural changes.

## Lymphatic system dysfunction

Lower-extremity lymphedema is a chronic disease that may develop after the treatment of gynecologic cancer. This condition may occur if pelvic lymph nodes are included in the scope of radiotherapy after pelvic lymph node dissection during surgery. It is of the utmost importance to detect and address lymphedema early, as it can be irreversible depending on its severity. The initial assessment often relies on self-reports; thus, it is essential that patients receive prior education on lymphedema. This education should cover prevention strategies, measurement techniques, and methods for massaging the lymph nodes.

Radiation oncology nurses play a crucial role in addressing the side effects caused by radiotherapy in clinical practice. Hence, they should be able to predict the likelihood of treatment-related adverse events based on a fundamental understanding of radiotherapy. They should also be educated in advance on how patients and their caregivers can respond to changes and self-manage, in addition to providing direct nursing interventions for adverse events. Furthermore, they should conduct evidence-based assessments, be knowledgeable of symptom management, and share the information with their teammates as part of a multidisciplinary approach. The role of radiation oncology nurses as patients’ supporters and educators will make a significant contribution to improving treatment adherence among patients undergoing radiotherapy.

## Conclusions

As radiotherapy techniques for gynecologic cancer become increasingly sophisticated and varied, the role of radiotherapy in cancer treatment is expanding. While the primary focus used to be on treatment outcomes, there is now a growing interest in the various issues experienced during the cancer survival period posttreatment, as well as symptom management during treatment. Despite the rising demand for care in tumor treatment, very few nursing curricula in Korea include education on radiotherapy, particularly the knowledge necessary for radiation oncology practice. Therefore, consistent efforts are needed to provide updated evidence-based practice, not only in clinical care but also in education.

## Figures and Tables

**Table 1.** Models for sexual assessment and intervention

ALARM Model	PLISSIT	BETTER	PLEASURE
Activity (sexual)	P– Permission	Bring up sexuality	Partner
Libido/desire	LI-Limited Information	Explain role of sexuality in QOL	Lovemaking
Arousal/orgasm	SS-Specific Suggestions	Tell about available resources	Emotions
Resolution/release	IT-Intensive Therapy	Timing critical	Attitudes
Medical data		Educate patient/partner	Symptoms
		Record in health record	Understanding
			Reproduction
			Energy

QOL, quality of life.
